# Biochemical changes and macrophage polarization of a silane-based endodontic irrigant in an animal model

**DOI:** 10.1038/s41598-022-10290-0

**Published:** 2022-04-15

**Authors:** Umer Daood, Muhammad Sharjeel Ilyas, Mariam Ashraf, Munazza Akbar, Ranjeet Ajit Bapat, Abdul Samad Khan, Mallikarjuna Rao Pichika, Abhishek Parolia, Liang Lin Seow, Suan Phaik Khoo, Cynthia Yiu

**Affiliations:** 1 Restorative Dentistry, School of Dentistry, International Medical University Kuala Lumpur, 126, Jalan Jalil Perkasa 19, Bukit Jalil, 57000 Bukit Jalil, Wilayah Persekutuan Kuala Lumpur Malaysia; 2 Department of Oral Biology, Post Graduate Medical Institute, 6 Birdwood Road, Lahore, Pakistan; 3 Department of Restorative Dental Sciences, College of Dentistry, Imam Abdulrahman Bin Faisal University, Dammam, 31441 Saudi Arabia; 4 Pharmaceutical Chemistry, School of Pharmacy, International Medical University, Kuala Lumpur, Malaysia; 5 Division of Oral Diagnostic and Surgical Sciences, School of Dentistry, International Medical University, Kuala Lumpur, Malaysia; 6 Paediatric Dentistry and Orthodontics, Faculty of Dentistry, The University of Hong Kong, Prince Philip Dental Hospital, 34 Hospital Road, Pokfulam, Hong Kong SAR, China

**Keywords:** Biological techniques, Drug discovery, Microbiology, Biomarkers, Materials science

## Abstract

Silane-based/fully hydrolyzed, endodontic irrigant exhibiting antimicrobial properties, is prepared, and is hypothesized to control macrophage polarization for tissue repair. Albino wistar rats were injected with 0.1 ml root canal irrigant, and bone marrow cells procured. Cellular mitochondria were stained with MitoTracker green along with Transmission Electron Microscopy (TEM) performed for macrophage extracellular vesicle. Bone marrow stromal cells (BMSCs) were induced for M1 and M2 polarization and Raman spectroscopy with scratch assay performed. Cell counting was used to measure cytotoxicity, and fluorescence microscopy performed for CD163. Scanning Electron Microscopy (SEM) was used to investigate interaction of irrigants with *Enterococcus faecalis*. K21 specimens exhibited reduction in epithelium thickness and more mitochondrial mass. EVs showed differences between all groups with decrease and increase in IL-6 and IL-10 respectively. 0.5%k21 enhanced wound healing with more fibroblastic growth inside scratch analysis along with increased inflammation-related genes (ICAM-1, CXCL10, CXCL11, VCAM-1, CCL2, and CXCL8; tissue remodelling-related genes, collagen 1, EGFR and TIMP-2 in q-PCR analysis. Sharp bands at 1643 cm^-1^ existed in all with variable intensities. 0.5%k21 had a survival rate of BMSCs comparable to control group. Bacteria treated with 0.5%k21/1%k21, displayed damage. Antimicrobial and reparative efficacy of k21 disinfectant is a proof of concept for enhanced killing of bacteria across root dentin acquiring functional type M2 polarization for ethnopharmacological effects.

## Introduction

Bacterial growth occurs inside infected root canals as well as deep within the dentinal tubules^1,2^. Bacteria are eliminated from these canals via a combination of mechanical debridement, irrigation, and intra-canal medicaments^3^. This preparation of the root canal reduces the population of microorganisms in addition to the adequate final shape allowing filling of the canal space^4^. Irrigant solutions like sodium hypochlorite (NaOCl) and chlorhexidine are pivotal disinfectants for the preparation of root canals. Previously, culture-dependant methods have traditionally been used to evaluate the antimicrobial effectiveness. However, it is almost impossible to remove the entire biofilm with current protocols, with remnants of bacterial growth observed against NaOCl in vitro studies^5^. In addition, there are complexities inside the root canal configuration which aid in the formation of biofilm bacterial colonies^6^. *Enterococcus faecalis* is one of the most important microorganisms producing intra-radicular infections^7^, surviving harsher environments of high alkalinity and antimicrobial resistance^8^.

Upon injury, macrophages have a huge contribution towards inflammation which eventually leads to healing of tissue and regeneration^9^. Macrophages can be divided into multiple subtypes depending on their surroundings and are commonly classified as M1 and M2 macrophages^10^. M1 macrophages are triggered by a Toll-like receptor during pro-inflammatory signalling, resulting in the release of proinflammatory cytokines such as IL-1 and 6 (IL-1, IL-6) and tumour necrosis factor (TNF)-α^11,12^. When tissue requires repair, the macrophage switches to M2 phenotype, which promotes anti-inflammatory actions while also stimulating the proliferation of fibroblasts, keratinocytes, and endothelial cells^13^. These pro-inflammatory mediators are crucial for the organism’s immune defence and for germ death. Continuous inflammatory activation in macrophages, on the other hand, may result in collateral tissue damage and chronic inflammation. Inflammation induced by macrophages is a hallmark of many pathologies, including infection, sepsis, and radiation sickness^14^. As a result, targeting M1 phenotype macrophages may open up new therapeutic avenues for inflammatory diseases.

Mitochondria are essential organelles in eukaryotic cells that regulate a wide range of biological processes, including ROS production, energy metabolism, stress response, and cell fate^15^. A growing body of literature has recently emphasised the importance of the mitochondrion as a key intracellular signalling platform that modulates innate immune and inflammatory processes^16^. Furthermore, sphingomyelin and cholesterol are important components of lipid rafts, which are lipid-rich microdomains in the plasma membrane that are important for signal transduction^17^. In addition, sphingolipids have been shown to elicit cellular inflammatory responses via a variety of molecular mechanisms, including the lipid raft mediated signalling mechanism in the plasma membrane. Sphingomyelin is required for lipid raft-associated receptor-mediated signal transduction. These sphingolipids are thought to activate cells directly via specific receptors^18^.

In this study, a novel antibacterial agent, Quaternary Ammonium Silane (QAS; KHG FiteBac Technology, Marietta, GA, USA), in the form of an endodontic irrigant is being used. QAS/k21 has a functional end-OH group that can be changed to initiate the –OH groups^5,19–21^. The antibacterial action is due to its –C18H37 lipophilic alkyl chain, which aids in bacterial penetration. In addition to broad-spectrum antibacterial activity with very low cytotoxicity, the k21 molecule elicits anti-MMP activity, which prevents further destruction of host tissues^21^. The newly developed antibacterial quaternary ammonium silane increased the resistance of dentin collagen to degradation by inhibiting endogenous matrix metalloproteinases and cysteine cathepsins^20^. In the presence of silanol Si–OH groups, the sol–gel process used to design silica-based materials undergoes a strong hydrolysis process, resulting in the release of alcohol molecules. Depending on the pH used, the condensation reaction has a direct effect on particle growth (sol formation) and aggregation (gel formation), with more alkaline conditions forming porous gels with dense networks^22^. Silica’s basic structure is similar to that of vitreous silica, which is a random network of SiO_4_ tetrahedral units arranged in cyclic, 4-ring siloxane structures. Thermodynamically, these structures are stable^23^. In our previous study, it was emphasised that the bacterial cells are lysed primarily through contact killing^24^, with k21 molecule absorbing on the cellular membrane, causing disorganisation and leakage of low molecular components. This results in a complete loss of cell structural organisation, impairing osmoregulation and other physiological functions^25^. The compound has functional end-OH groups on its surfaces that can be activated by acids. The experimental endodontic irrigant is hypothesised to have long-lasting antimicrobial efficacy due to quaternary ammonium molecules and their covalent attachment in the presence of silanol groups. In addition, the compound in combination with nano-polylactic glycolic acid and riboflavin, exhibited superior antibacterial/antibiofilm effects against cariogenic biofilms after bonding-resins infiltration without adversely affecting bond strength^26^. Moreover, favourable antimicrobial and endodontic profile of the sodium hypochlorite and 2% quaternary ammonium silane solution exhibited predictable reduction of intracanal bacteria^27,53^.

In this study, we fabricated an injectable silane-based endodontic irrigant that exhibits antimicrobial properties. This engineered irrigant solution is hypothesized to control macrophage polarization for tissue repair and regeneration. Despite our previous study describing various antimicrobial and transdentinal macrophage activities^28^ mediated by the quaternary ammonium compound, no in-depth studies have been conducted to assess the compound’s ability to modulate macrophage polarization. The aim of the study was to evaluate that k21 irrigant could change macrophage polarization because macrophages modulate the ethnopharmacological effects of polarization. More importantly, the underlying molecular mechanisms were thoroughly investigated. Our findings shed new light on the role of mitochondrial mass in the process of macrophage proinflammatory differentiation, which will aid in the treatment of a variety of inflammatory diseases, including endodontic infections. Nowadays, research is focusing on developing anti-inflammatory agents with selective pharmacology and low toxicity. Based on the foregoing, the study was designed to assess QAS/k21 anti-inflammatory effect in vivo. The null hypothesis tested was, k21 irrigant could not change macrophage polarization which will aid in the treatment of inflammation.

## Results

**Figure 1:**
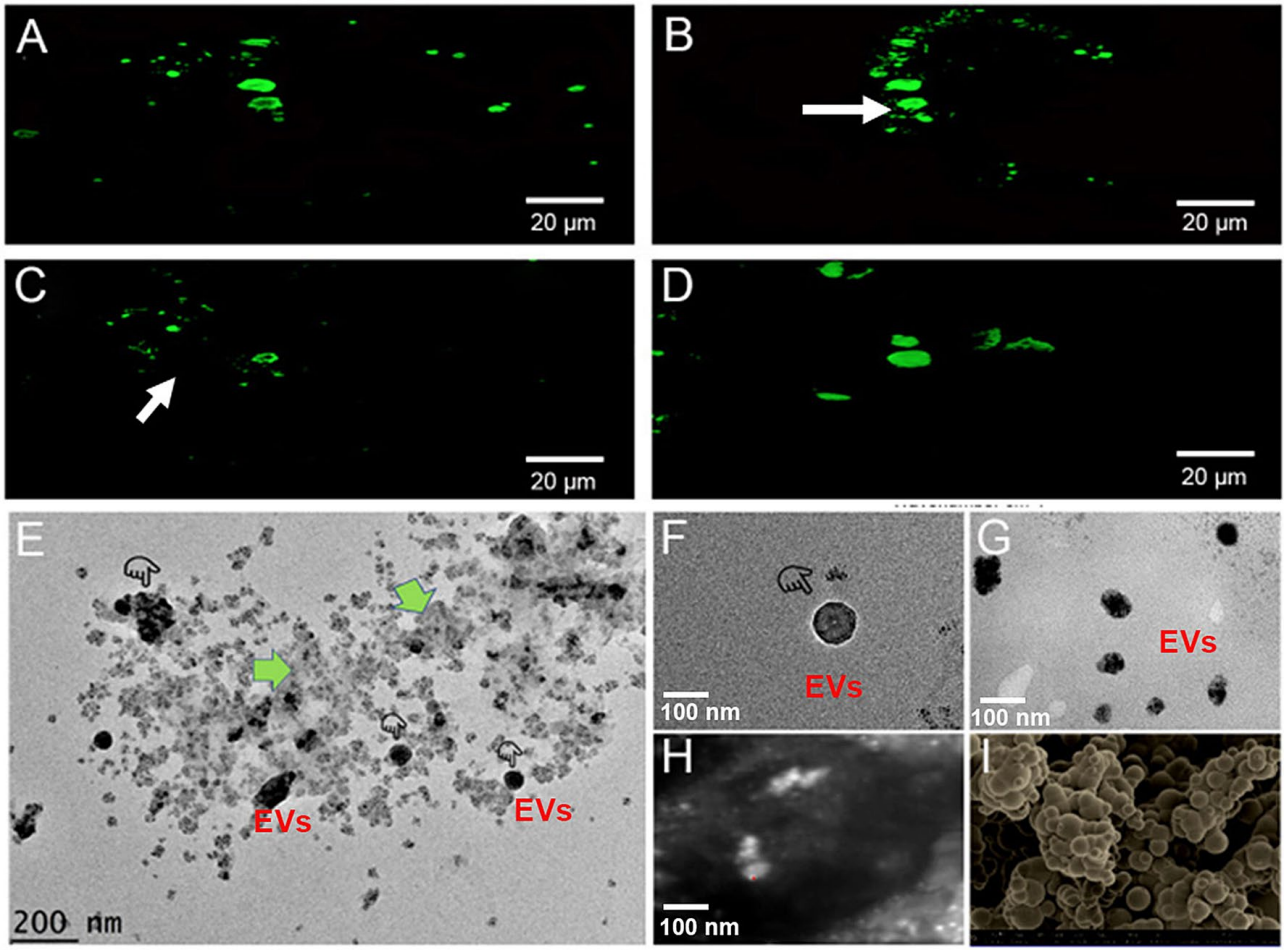
Laser scanning confocal microscopy was used to examine mitochondrial morphology in macrophages stained with MTG after exposure to different irrigant solutions. The magnification changer was set to 1.6 × zoom (pixel size = 0.1667 μm) or 1 × zoom (pixel size = 0.2667 μm). The white arrows indicate the continuity and discontinuity in the mitochondrial mass. (**A**,**B**) 0.5% k21 treated macrophages had more mitochondria with typical morphology when compared to resting cells of (**C**) control and (**D**) 1%k21. (**E**,**F**) The distribution of exosomes was evident in 0.5%k21 and (**G**) 1%k21 specimens; (**H**) high magnification image through confocal microscopy showing presence of mitochondria in 0.5%k21 specimens; (*I*) presence of fully hydrolyzed and condensed k21 particles.

Laser scanning confocal microscopy was used to examine mitochondrial morphology in macrophages stained with MTG after exposure to different irrigant solutions (Fig. 1). The experiments confirmed that MitoTracker accumulates in mitochondria and increases fluorescence when accumulated inside the organelles. The 0.5% k21-treated macrophages had more mitochondria with typical morphology when compared to resting cells of the control and 1%k21 groups. Although the differentiated macrophages following treatment with 0.5%k21 had a larger cell shape and more mitochondria, cell size does not always correlate with mitochondrial size. Ultrastructural analysis consistently revealed that mitochondria in the 0.5%k21-treated macrophages had greater mitochondrial mass, shorter length, and lesser cristae than those in control macrophages (Fig. 1A–C). Similar features were observed in the 1%k21-treated cells, although low power reconstruction displayed less stained mitochondria in few cells treated with 1% k21 irrigant. (Fig. 1D).

The distribution of exosomes showed no difference between the control, 0.5%k21, and 1%k21 groups as shown in Fig. 1. In the specimens from the control group, no vesicles were observed to be released. M0, M1, and M2 macrophages were used to isolate the cells. The size of vesicles corresponds to the size distribution of internal exosomes in control and k21 specimens. The established cytokine-based protocols induced macrophage polarization along with IL-6 expression in M1 polarized cells, validating cytokine stimulation acquired due to known macrophage characteristics.

**Figure 2:**
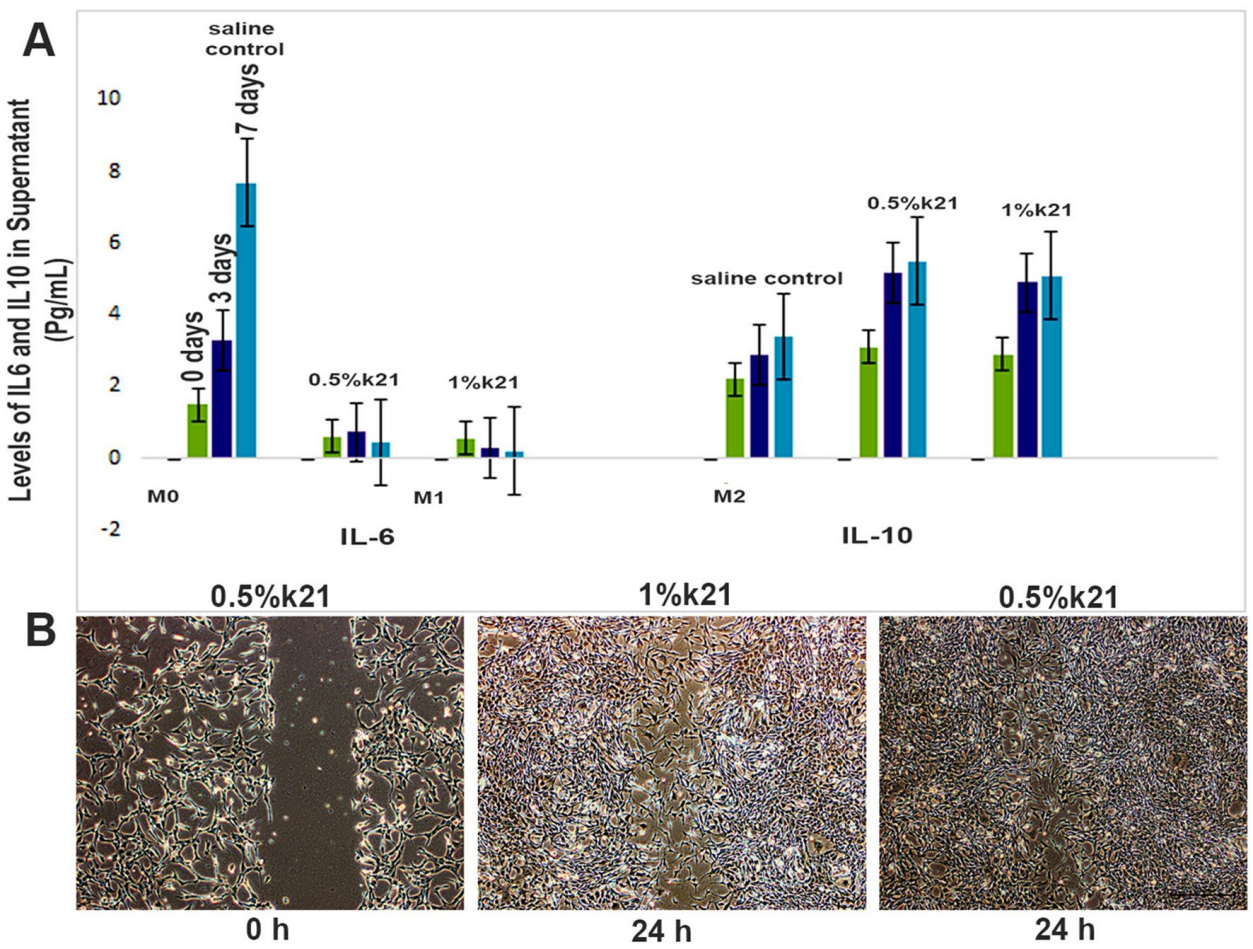
(**A**) Effect of k21 on the synthesis and intracellular levels of IL-6 and IL-10 in macrophages for M1 and M2 polarization. Under different specific microenvironmental stimuli and signals provided, macrophages produce distinct functional phenotypes as a reaction and polarized into classically activated pro-inflammatory (M1), non-activated (M0) and alternatively activated anti-inflammatory (M2) macrophages. *p < 0.05. (**B**) Photomicrographs of the wound treated with vehicle (DMSO) and k21 groups after 24 h.

There was a significant decrease in IL-6 supernatant levels expression in k21-treated cells when compared to control cells (Fig. 2).

The mean supernatant levels of IL-6 in k21-treated cells were significantly lower at 0.6 pg/mL (baseline) and 0.5 pg/mL for 0.5% and 1% k21 groups, respectively at baseline. Similar differences were observed after 3 and 7 days. The proportion of intracellular expression is considered significantly lower in k21-treated cells. To identify macrophages of the M2 phenotype, IL-10 markers were found to be more abundant in k21 polarized cells, especially 1%k21-treated cells than in control cells (Fig. 2) after baseline, 3 and 7 days. The IL-10 was found to be significantly higher in 0.5% and 1% k21 groups. The levels of IL-10 secreted by macrophages treated with 1%k21 were 2.9 pg/mL and 5.1 pg/mL at baseline and 7 days, which were significantly higher than those secreted by control polarized cells (*p* < 0.05).

**Figure 3:**
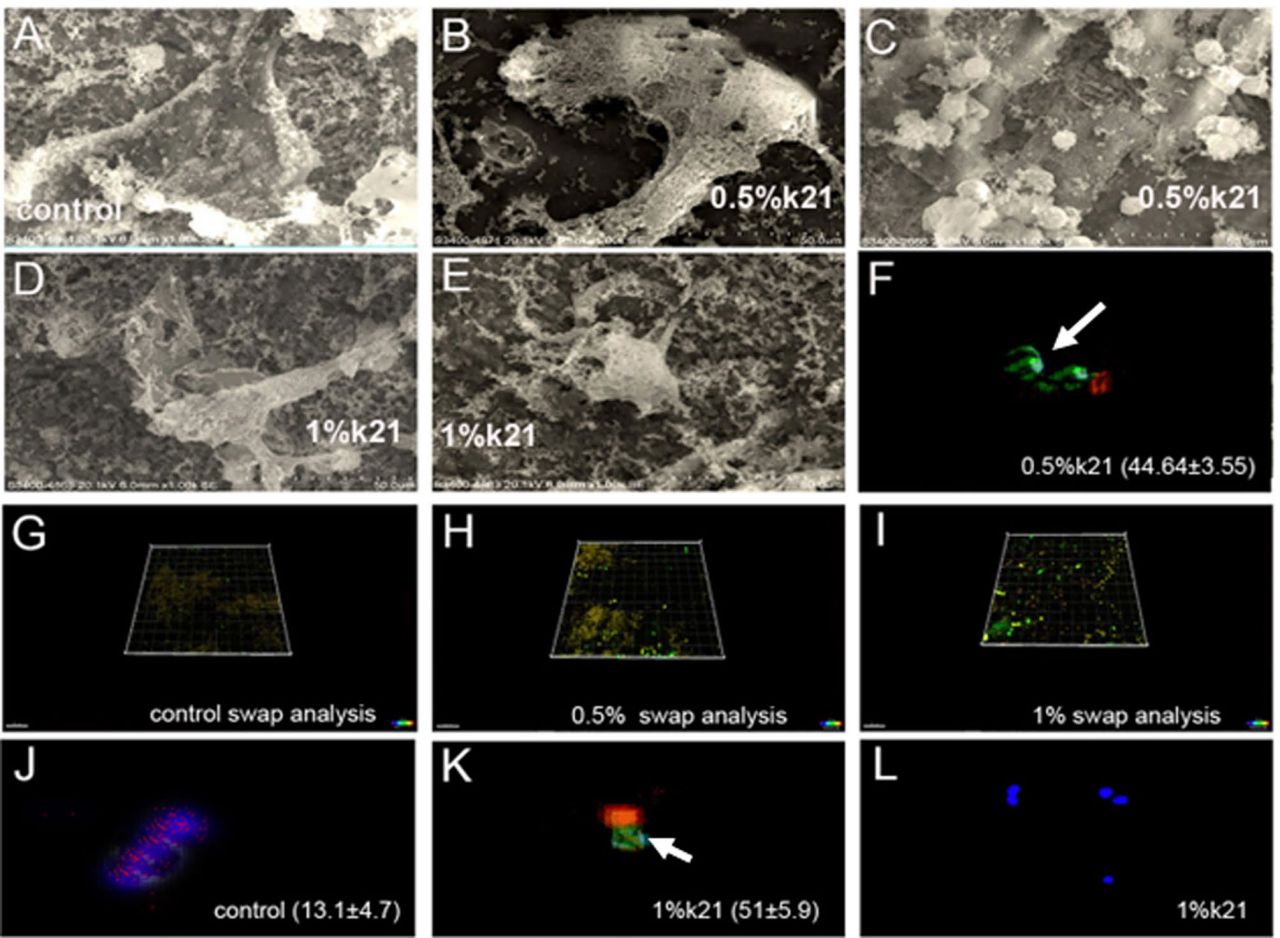
The viability results of BMSCs. According to the green fluorescence staining in the cell layer after 24 h, most cells were viable in all experimental groups as per SEM analysis (**A**–**E**). BMSCs treated with a modified medium ok 0.5% k21 concentrations (Fig. 5B,C) had a survival rate comparable to the control group (Fig. 5A). Cells suffered more cytotoxicity at 1% k21 concentrations reducing their viability slightly (Fig. **D**,**E**). (**F**–**L**) The immunofluorescence showed a significantly higher percentage of M2 polarized macrophages with over expression of CD163 markers (*p* < 0.001) for 0.5%k21 and 1%k21 specimens. White arrows indicate green fluorescence staining depicting viability.

The photomicrographs of the cells were captured at 0, 18, and 24 h after making the scratch and are shown in Fig. 3.

The wound area (mm^2^, *n* = 9) and wound closure (%, *n* = 9) are expressed in mean ± S.D. and shown in Table 1.

**Table 1:** The effect of 0.5% and 1%k21 on wound area (mm^2^) and wound closure (%).

Time (h)		0	12	18	24	Relative cell viability
Wound area (mm^2^)	Vehicle	3.27 ± 0.14	2.76 ± 0.19	2.36 ± 0.32	2.12 ± 0.11	
	0.5%k21	3.88 ± 0.12	1.69 ± 0.13	1.27 ± 0.14	0	
	1%k21					
Wound closure (%)	Vehicle	0	15.61 ± 3.24	27.81 ± 5.56	35.13 ± 4.12	53.4% ± 0.99
	0.5%k21	0	57.44 ± 3.26	68.17 ± 3.14	100	79.9% ± 0.8
	1%k21	0	44.5 ± 4.3	51.3 ± 6.6	78.1 ± 5.1	63.4% ± 0.5
						µg/mL

The photomicrographs in Fig. 2B show the progress of wound healing between the untreated and treated wounds at time points 0, 12, 18 and 24 h. It is suggestive that 0.5%k21 enhanced the wound healing activity as no visible wound area was visible at the 24 h when compared to the vehicle control (0.1% DMSO). The qPCR analysis (Table 2) showed that wound healing markers were upregulated. The 0.5%k21 group was found to have upregulated the inflammation-related genes, ICAM-1 (3.55 ± 0.29), CXCL10 (2.85 ± 0.14), CXCL11 (2.62 ± 0.41), VCAM-1 (2.27 ± 0.23), CCL2 (2.00 ± 0.11), and CXCL8 (1.60 ± 0.04); tissue remodelling-related genes, collagen 1 (2.91 ± 0.03), EGFR (2.01 ± 0.08) and TIMP-2 (0.94 ± 0.01); and immune modulation-related gene, M-CSF (1.01 ± 0.11). Out of the panel of genes tested, treatment with 0.5%k21 resulted in a significant upregulation of ICAM-1 and collagen-1. These results coincide and support the wound healing activity observed from the in vitro scratch assay.

**Table 2:** List of primers used in quantification of genes by qPCR.

Gene	Forward primer (5’)	Reverse primer (3’)
CCL2	AGTAGGCTGGAGAGCTACAA	GTATGTCTGGACCCATTCCTTC
VCAM-1	GCACTCTACTGCGCATCTT	CACCAGACTGTACGATCCTTTC
ICAM-1	CCAGTACTGCTGGTCATTGT	TCCTCCTGAGCCTTCTGTAA
Collagen I	AGACCTGTGTGTTCCCTACT	GAATCCATCGGTCATGCTCTC
CXCL10	AGTAACTGCCGAAGCAAGAA	GCACCTCCACATAGCTTACA
CXCL11	TTCCTGTGAGTCTGCCTTTG	CAGCCATCCCTACCATTCATT
CXCL8	TACCATCCAGACCAGAGTCA	GGACGAAGATGCCTAGGTTAAG
EGFR	ACAGCGCTACCTTGTTATCC	CATCCTCCATGTCCTCTTCATC
M-CSF	CAGGTGGAACTGCCAGTATAG	GAAGATGGTAGGAGAGGGTAGT
TIMP-2	CCATGATCCCTTGCTACATCTC	TGCCCATTGATGCTCTTCTC
GAPDH	AACAGCAACTCCCACTCTTC	CCTGTTGCTGTAGCCGTATT

**Figure 4:**
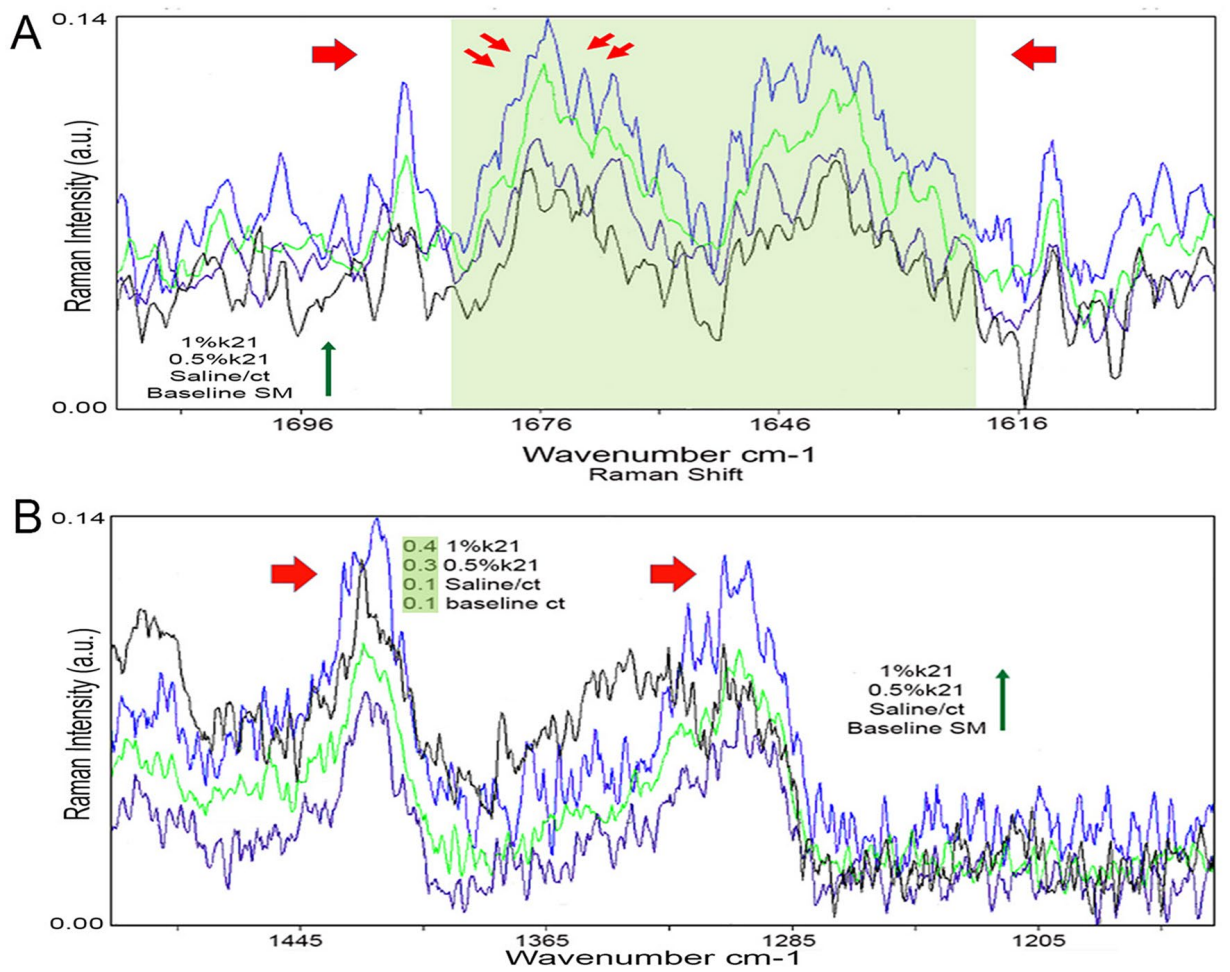
Intensity variations of the Raman spectrum of sphingomyelin presence within the macrophage cell suspension and a spectral comparison. All samples were deposited on the glass-bottomed dish and measured at 23 °C. (**A**) A shoulder visible in the spectrum evolved into a sharp band at 1643 cm^-1^ that existed in all groups but at different intensities. The two bands observed at 1672 and 1642 cm^-1^ and 1440–1445 are assigned to the stretching modes of the hydrogen-bonded amide group and CH2 deformation, respectively (Fig. **A**,**B**). The amide group’s C = C and C = O stretching mode contributes primarily to the hydrogen bonded amide I band which are at higher intensities in k21 groups as compared to the control (*p* < 0.05), including the CH_2_ twist at 1301 cm^-1^ (Fig. 4B). Different groups are represented by different colours.

The Raman spectra of cell suspensions are shown in Fig. 4. The bottom and top spectra were measured after sample deposition on a glass bottomed plate. The origin of the 1643 cm^-1^ band is not well understood. Table 3 shows the tentative assignments of the main Raman bands of the sphingomyelin groups.

**Table 3:** Assignment of the main raman bands for sphingomyelin.

Raman shift cm^-1^	Assignments
1643	Amide I band
1445	CH_2_ deformation
1301	CH_2_ twist
1135	C–C stretching
961	CN asymmetric stretching
881	acyl C1-C2 stretching

**Figure 5:**
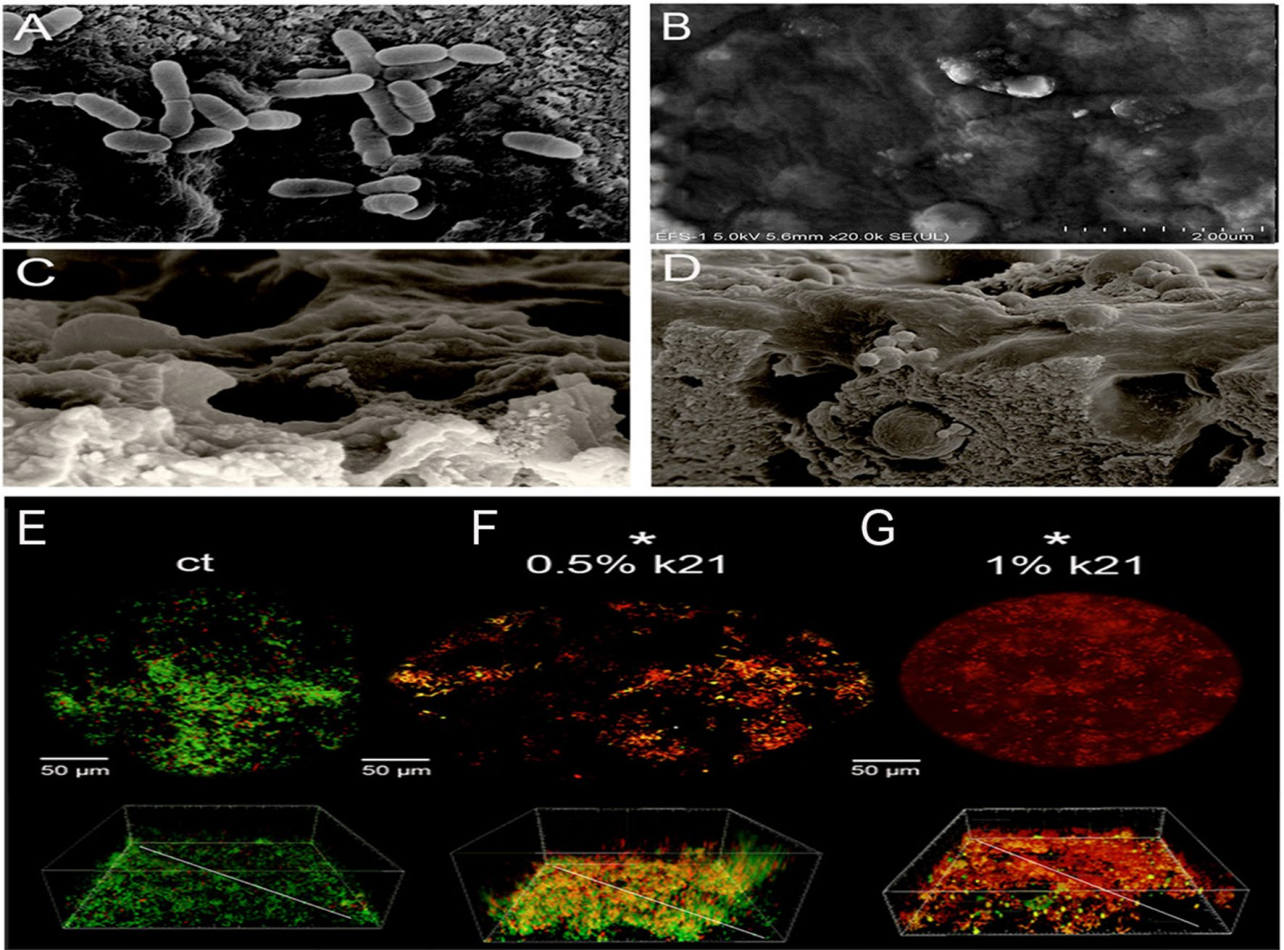
Display of typical morphologies of *E faecalis* after antimicrobial treatment. The bacteria treated with saline (**A**) remained intact and smooth, whereas the bacteria treated with 0.5%k21 (**B**), especially 1%k21 (**C**), displayed damaged and removed bacteria from the dentin substrate. Maximum detachment was seen in 1% groups (**D**). CLSM images that are representative of E faecalis species depicts the control specimens in BHI medium (**E**) showed clusters of green colonies as the majority of the dead bacterial cells formed large aggregates and were found in 1% (**F**) and 0.5% QAS specimens (**G**).

A shoulder visible in the spectrum evolved into a sharp band at 1643 cm^-1^ that existed in all groups but at different intensities (Fig. 4A). The spectrum is a hydration-sensitive band at 1643 cm^-1^ which is significantly higher in the k21 groups as compared to those of control. The hydrogen bond of the amide groups produces a band at 1650 cm^-1^ (data not shown).As a result, the two bands observed at 1672 and 1642 cm^-1^ and 1440–1445 are assigned to the stretching modes of the hydrogen-bonded amide group and CH2 deformation, respectively (Fig. 5A,B). The amide group’s C = C and C = O stretchingmode contributes primarily to the hydrogen bonded amide I band which are at higher intensities in k21 groups as compared to the control (*p* < 0.05), including the CH_2_ twist at 1301 cm^-1^ (Fig. 4B).

**Figure 6:**
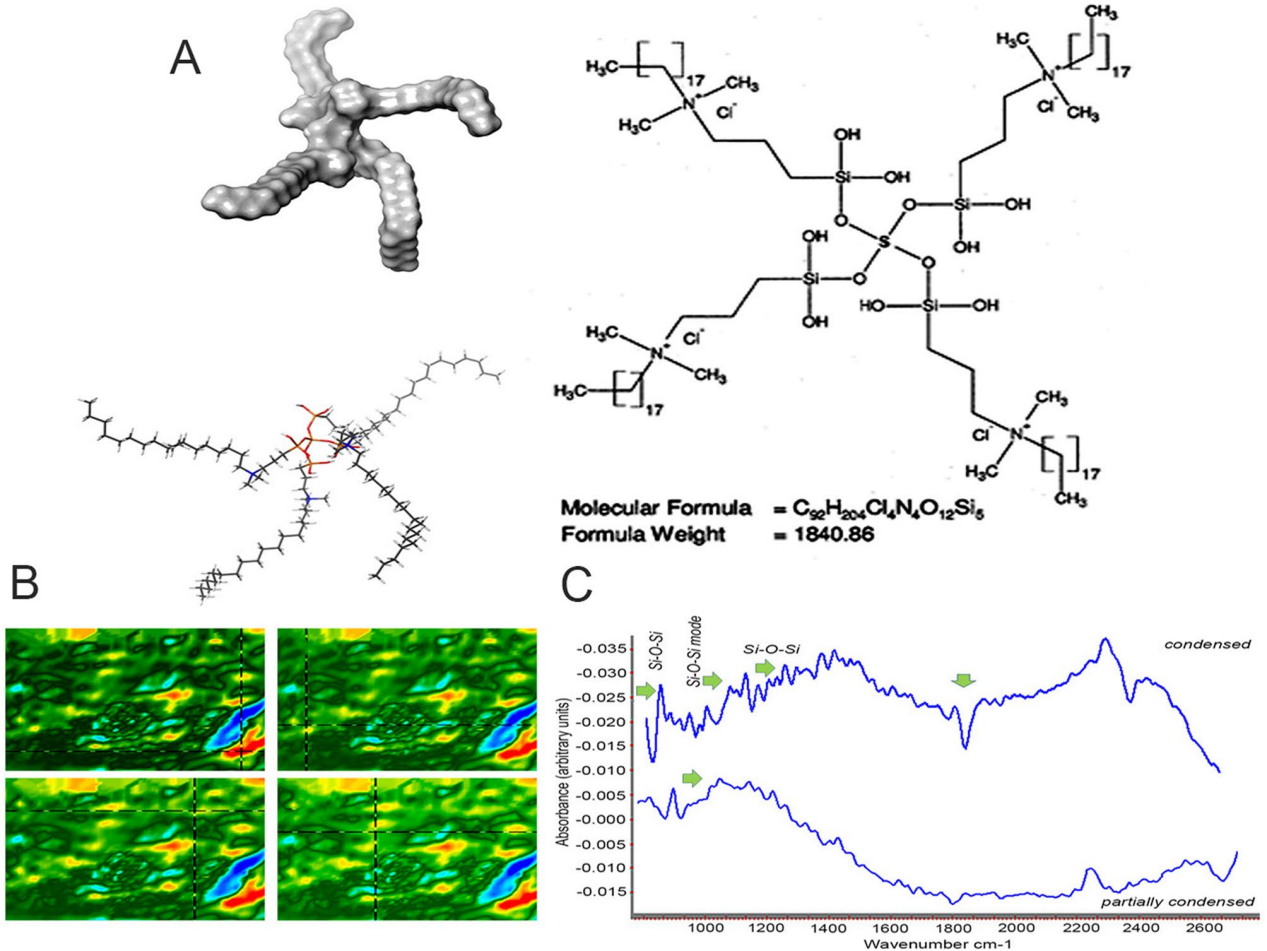
(**A**) Chemical formula and 3D simulation of fully hydrolyzed Quaternary Ammonium Silane (ethanol-based solution form; k21 0.5% and 1%) prepared by sol–gel synthesis, with SiQAC molecule substituted with 3-(triethoxysilyl)-propyldimethyloctadecyl ammonium chloride (i.e., the ethoxy version of SiQAC, abbreviated as Et-SiQAC) and coupled with tetraethoxysilane. (**B**,**C**) Fourier transform infrared spectroscopy of fully condensed k21/E QAS solution with ethanol as the reference, alongside inset heat maps of the solution. The hydrolysis reaction is between the tetraethoxysilane and Et-SiQAC and characterized by the presence of silanol groups. FTIR depicts full reaction with absence of any contamination.

The viability results of BMSCs are shown in Table 1. According to the green fluorescence staining within the cell layer after 24 h, most cells were viable in all experimental groups (Fig. 3A–E). Interestingly, some cells were clearly visible while others were only vaguely visible, indicating that cells were distributed uniformly across the layers and could survive in the deeper layer as well. BMSCs treated with a modified medium of 0.5% k21 concentrations (Fig. 3B,C) less than 50 g/mL had a survival rate comparable to the control group (Fig. 3A). Cells suffered more cytotoxicity at 1% k21 concentrations reducing their viability slightly (Fig. 3D,E). The immunofluorescence showed a significantly higher percentage of M2 polarized macrophages with over expression of CD163 markers (*p* < 0.001) for 0.5%k21 (Fig. 3F/H) (44.64 ± 3.55) and 1%k21 (51 ± 5.9) specimens (Fig. 6I/K/L) as compared to control (Fig. 3G/J). The results were in correspondence to the SEM evaluation which showed no morphological changes after antimicrobial treatment.

Figures 5 displays the typical morphologies of *E. faecalis* after antimicrobial treatment. The bacteria treated with saline (Fig. 5A) remained intact and smooth, whereas the bacteria treated with 0.5%k21 (Fig. 5B), especially 1%k21 (Fig. 5C), displayed damaged bacteria which were removed from the dentin substrate. Figure 5A on the other hand, shows the deposition of thick aggregates of bacteria within the control specimens. The groups left single or multiple deposits on the sample, with bacterial cells clumping and chaining together to form complex bioflms. After treatment with 0.5% and 1% k21, dramatic changes in the bioflms were observed. Due to slight restructuring, there were no colony chain formations among 0.5%k21 specimens (Fig. 5B,C), in addition to maximum detachment seen in the 1%k21 group (Fig. 5D). CLSM images that are representative of *E. faecalis* species depicts the control specimens in BHI medium (Fig. 5E) showed clusters of green colonies as the majority of the dead bacterial cells formed large aggregates and were found in 1% (Fig. 5G) and 0.5% k21-treated specimens (Fig. 5F). The concentration and type of disinfectant used had an effect on aggregation (*p* < 0.05). In contrast to the control specimens, the k21 specimens exhibited different characteristics, forming thinner bioflms with no distinct aggregates, indicating a dominant role for k21 in bacterial biofilm removal and non-adherence (Table 4) The computed live bacterial proportions for both *S. mutans* and *S. sanguinis* with k21 treatment was significantly lower than the control group (*p* < 0.05). The least dead bacterial proportions were observed with the control (NaCl) group followed by 0.5% k21 treatment (*p* < 0.03).

**Table 4:** Bacterial live/dead proportions in mono-species biofilms.

Groups	Live (%)—Mean (SD)	Dead (%)—Mean (SD)
*E. faecalis*		
Control	99.1 (10.1)^a^	0.9 (0.1)^1^
0.5% arg	29.5 (5.1)^b^	70.5 (7.0)^2^
1% arg	4.8 (1.4)^c^	10.8 (4.8)^3^

Different superscript lowercase (a, b, c)/uppercase (A, B, C) English alphabets, numbers (1, 2, 3), represent significant differences between different treatment groups

1-way ANOVA with Turkey’s HSD post-hoc test; *p* < 0.05 is significant

## Discussion

The ability to resolve inflammation is critical in wound healing^29^. Several cytokines associated are expected to contribute to macrophage to switch from M1 to M2; TNF-α and IFN-γ promote M1 polarization along with LPS, through activation of the NF-*k*B and MAPK pathways^30^. We hypothesised that k21 could influence macrophage activation and differentiation in this study. To assess this effect, we first looked into whether different concentrations of k21 solutions had any effect on macrophages. Our findings may shed light on a novel mechanism involved in these biological effects, specifically an increase in the rate of M2/M1 responses. M2 polarization plays an important role in tissue regeneration during wound healing by producing soluble factors such as IL-10 and TGF-beta, which increase angiogenesis and collagen production by fibroblasts while inhibiting the inflammatory process^31^. The capacity of k21 specimens to promote M2 differentiation suggests further evaluation of the role of macrophages. The discovery of new molecules (k21) with the potential to regulate macrophage polarization could lead to new therapeutic strategies for a variety of endodontic problems. In addition, our previous study confirmed the presence of these molecules as the predominant compounds^27^, which has been described for its anti-inflammatory activities. However, little is known concerning their immunomodulatory properties, and further studies are desirable. Additionally, we cannot disregard the fact that the effect of k21 is due to the synergism of these molecules that were not yet evaluated for their biological effects.

New evidence suggests that mitochondria may serve as an important intracellular signalling platform for regulating immune responses^32^. The study results show that pro-inflammatory differentiated macrophages, those especially treated against saline have less mitochondrial mass than cells tested against k21 groups in this study. It is noteworthy to mention that LPS causes significant macrophage spreading and enlargement, which is consistent with previous findings^33^. Meanwhile, k21 inhibition effectively improved mitochondrial mass in LPS-treated macrophages. We concluded that mitochondrial mass in LPS-activated macrophages was related to pro-inflammatory cytokine production which was inhibited in case of k21 solutions. The balance of fission and fusion determines mitochondrial mass. Increased fusion promotes adjacent mitochondrial integration, whereas increasing fission promotes mitochondrial fragmentation^34^. Furthermore, k21 significantly improves LPS-induced mitochondrial mass increase and macrophage M1 differentiation in vivo and in vitro^27^. However, the role of mitochondrial biogenesis in regulating the inflammatory response remains debatable and contrary to previous findings. Previous research, for example, has shown that increased mitochondrial biogenesis aids in inflammation resolution^35^. The results of mitochondrial ultrastructure revealed that a low concentration of k21 had little effect on the mitochondria. The destruction of the mitochondria would be accompanied by the injury of the respiratory chain, confirming that the respiration rate would improve in presence of k21 groups at lower concentrations. Quaternary ammonium compounds have the structure of lipophilic cations, which are known to be preferentially taken up by mitochondria^36^. This indicates a possible structure activity relationship in context to the mitochondrial effects of k21 groups, which needs detailed investigation. A detailed comparison of the antimicrobial efficacy and mitochondrial inhibitory effects of the k21 groups needs to be performed in order to identify the quaternary ammonium compounds with high antimicrobial efficacy with minimum mitochondrial effects especially when known pharmacologically relevant benzalkonium chlorides inhibit mitochondrial ATP production and oxygen consumption in human cells^37^. Moreover, toxic action of NaOCl on mitochondrial function is produced after the toxic agent interacts with the cellular membrane or other molecules of the matrix, causing loss of cell adherence to the culture surface^38^. Hypochlorite is more potent in inhibiting macrophage adhesion^39^, indicating the fact that this loss of cell adherence could be considered as the first sign of toxicity. Furthermore, the ability of cells to overcome NaOCl-induced damage could not be confirmed through our experiments.

Following monocyte/macrophage depletion, the immunomodulatory role of macrophages in bone repair and regeneration is clearly observed^40^. While the role of macrophages in bone repair immunomodulation is now well established, the current findings suggest that EVs and their cargo play an important role in mediating the contrasting effects of polarised macrophages on regeneration. EVs have also been shown to play a role in immunomodulation in the context of osteoblast regulation of osteoclastogenesis^41^. While previous research has concentrated on cytokine, growth factor, and chemokine phenotypes, EVs and their cargo may be useful phenotypic markers of macrophage polarization^42^. The average particle size, and exosomal marker expression of the EVs were not affected by macrophage polarization. The ability of EVs from naive and polarised macrophages to be endocytosed by MSCs remained unchanged, with a dose-dependent and saturable endocytic profile. This study builds on these findings by demonstrating that isolated macrophage EVs, which are devoid of other secretome constituents, also contributing to osteoinduction and bone regeneration in vivo.

There was a significant decrease in IL-6 supernatant levels expression in k21-treated cells when compared to control cells at baseline and after 7 days (*p* < 0.05). These results suggest that k21 groups have anti-inflammatory capacity in M1-primed cells. This anti-inflammatory effect against IL-6 can be protective in inflammatory mediated endodontic diseases. The same study also observed k21 effects in the animal model against IL-10. Our findings suggest that k21 can reduce chemotaxis to injured tissues by impairing macrophage sensitization to chemokines. In chronic inflammation, excessive macrophage migration to the inflammatory site may impair tissue regeneration. The removal of dead and fragmented cells is an important step in wound healing because it helps to resolve the inflammation process and, as a result, tissue repair. This process is mediated by upregulation in M2 macrophages. Furthermore, M2 macrophages aid in tissue repair by producing anti-inflammatory cytokines like IL-10^43,44^. Based on these findings, k21 was found to modulate macrophage functional polarization into the M2 phenotype. The suppression of IL-6 could be caused in part by specific changes in cell activity. The lipid bilayer may act as a reservoir for lipophilic/amphiphilic ligands, which helps to concentrate the ligands near the receptor's binding site, influencing the observed association rate without changing interactions with binding-site residues. Therefore, it is speculated that the k21 concentrations are well tolerated in lipid bilayers at the specific concentrations. Because re-epithelialization and granulation tissue formation are two important factors in wound healing^45^, it was necessary for us to measure the scratch analysis. In addition, ICAM-1 is an endothelial and leukocyte-associated transmembrane protein that has long been recognised for its role in cell–cell interactions and leukocyte endothelial transmigration^46^. Because of its associations with immune responses, it has been proposed that ICAM-1 may play a role in signal transduction.

The spectra amongst the 1643, 1676 and 1445 cm^-1^ are almost identical showing changes in the Raman spectra within the expected regions. Shoulders were seen within the spectrum which gradually evolved into sharp bands. The Raman analysis at 1643 cm^-1^ revealed a hydration sensitive band corelated with a water content. The broad feature at 1644 cm^-1^ is designated to the Amide I band which has a strong association of amide linkage towards the acyl chain and amino group of sphingosine base. The 1644 cm^-1^ feature is also associated with the HOH deformation mode of water molecules associated with the bilayer hydrogen-bonding network^47,48^. In essence, the characteristic shift in the position of the amide I band observed in sphingomyelin Raman spectra reflects SM's degree of intermolecular hydrogen bonding. All spectra in k21 groups had shown increased intensities as compared to other groups. SM is a sphingolipid (SL) family membrane lipid that has been found in macrophages^49–51^. The sphingo lipids are produced in the endoplasmic reticulum and the Golgi but accumulate in the cell membrane. Transport vesicles transport material from the site of synthesis in the peri-nuclear region to the membrane. By apical budding, SLs self-assemble at the trans face of the Golgi apparatus to form vesicles with high SL concentrations, which are then transported to the cytosolic surface^52^. SLs are essential for cell recruitment and phagocytosis of foreign material, and their inhibition has a significant impact on the clearance of microorganisms/particulate material^53^. The Raman spectrum within the sphingomyelin regions can be visualized to be rich in SM for the k21 groups. With the higher intensities, the sphingomyelin may be associated with the active transport of SM to the cellular membrane playing a pivotal role in the phagocytic function of the macrophage cell. This was seen in the vesicle excretion studies corresponding to internal exosomes with similar size distribution for k21 specimens^54^.

There were minimal dead cells, indicating that this endo delivery system was not toxic for encapsulating BMSCs. The current study compared the ex vivo biocompatibility of k21 groups and saline groups after 24 h of contact with BMSCs. According to the results of the cytotoxicity assay, a higher percentage of cells remained viable after being exposed to 0.5%k21 versus 1% k21 groups. As a result, the first null hypothesis, that no difference in cytotoxicity exists between k21 and other groups, must be rejected. This renders the k21 solution a safer disinfectant for incorporation inside teeth substrate. More research is needed to determine the mechanisms underlying k21 improved biocompatibility and evaluation of accidental extrusion of the irrigant to peri-apical tissues to evaluate adverse inflammatory reaction.

The immune cell state is indispensable for regeneration, tissue development and repair^55^. The indwelling of biomaterials, k21 in this case, can initiate a series of immune responses for active host defence and tissue repair. Amongst the many cells, macrophages have received considerable attention for better tissue modelling outcomes. Changes within the macrophages are related to the polarization states as the cells seeded appeared to have different shapes when treated with k21 irrigant in contrast to cells seen amongst the control groups. The trend was more apparent for the altered expression of specific markers of macrophage polarization, CD163.

The histological analysis, the number of inflammatory cells verified by M2 immunochemistry (higher concentrations of IL-10) is indicative of favourable effect. However, the exact molecular mechanism is an important area for future investigation because the concentration of k21 might have been more at the contact surface, but there is no way to test this possibility to date. This will be an area of interest for the authors.

Bacteria from the endodontic instruments and anatomic complexities are thought to be a permanent source of reinfection^56^. Prior to evaluating the efficacy of the experimental irrigant, a standardized single species biofilm model was established in the current study. The use of a tetrafunctional organosilane as the anchoring unit for the antimicrobial trialkoxysilane molecules in the k21 formulation allows for the formation of a three-dimensional network once condensation is completed within the dentinal substrate. Contact inhibition^57^ causes *E. faecalis* membrane damage, resulting in lysis. The cationic surfactant class is distinguished by a positively charged hydrophilic head and an alkyl-based hydrophobic tail with an aliphatic salt's central nitrogen atom. The quaternary ammonium silane molecule attaches to the bacterial cell wall, causing reactions with cell wall proteins and lipids that result in structural wall disorganisation, nucleic acid degradation inside the cell, and the release of autolytic enzymes of cell components^58,59^. The quaternary ammonium silane is expected to penetrate the phospholipid acid-based hydrophobic membrane core, increasing surface pressure, and causing membrane transformation. This transformation is a direct result of the transition from liquid-to-liquid crystal state. This reduces the hydrophobicity of the bacterial membrane core by converting the phospholipid building blocks of the bacterial membrane into hexagonal structures^55^. In our experiments, 0.5 percent k21/E caused a dose-dependent disruption of bacterial colonies, indicating that cationic quaternary ammoniums were at work. It has previously been reported that the demonstrated antimicrobial activities are based on amphiphilicity ^21^. The hydrolysis reaction within the molecule takes place between tetraethoxysilane and Et-SiQAC, which is distinguished by the presence of silanol groups. The FTIR spectrum revealed the complete presence of silanol groups, ethanol, and water, with the Si–O–Si open chained species identified as impurities in the partially condensed version of 0.5 percent k21-E (Fig. 6). The partially condensed version was created because it can be dissolved in ethanol, whereas the optimally condensed version of the k21 is insoluble in the solvent. The study of biofilms morphology was also previously performed. NaOCl eliminated all the *E. faecalis* cells adhered leading to to an almost complete loss of living cells with a predominance of pear-shaped cells, when compared with the morphology of the untreated mixed biofilm^60^. However, current investigations and previous studies highlighted potential advantages of K21 as an irrigation solution with significant root canal disinfection as compared to NaOCl with no regrowth of biofilm complexes^5,54^.

There are several factors like large surface area and tubules within the root canal system and a compacted smear layer that can reduce the effectiveness of an irrigation solution. In our study, we have not yet established the mechanical effect on canal walls, stress and the force exerted on the canal surface by the k21 flow. Endodontic infections are polymicrobial and any analysis based on such biofilms has better results on determining any antimicrobials potency. However, the *E. faecalis* model is chosen for its ability to invade dentinal tubules^61^, and its frequent isolation from apical periodontitis cases^62^. In addition, the organism has shown high survival rates in starvation, high pH levels and entombment^63,64^. Therefore, multispecies model work is currently underway test the efficacy of k21 against complex nature of endodontic infections. In addition to this, the volume of k21 irrigant used in the animal model is a limitation which may not reflect the clinical amount used in dental clinical practice. The choice of choosing disinfection and macrophage polarization is limited. To overcome the aforementioned limitations, many methods have been combined to assess spatially the killing performance and modulating inflammation. k21 quaternary ammonium silane is impermeant and destroys bacterial colonies based on cell membrane damage. However, the methods do not measure metabolic activity which is under focus for optimizing the formulation detailing the effects of this novel endodontic irrigant.

## Conclusion

Altogether, our study reports the antimicrobial and reparative efficacy of k21 disinfectant which is a proof of concept for enhanced killing of bacteria across the root dentin. The material is biocompatible and is currently used in Food and Drug Administration approved formulations for coronal cavity disinfection (2%k21 antimicrobial cavity cleanser; Fitebac; aqueous ethanol solution). This efficacy should be further investigated using clinical trials for an efficient endodontic treatment.

## Materials and methods

### k21 Irrigant solutions

**Figure 7:**
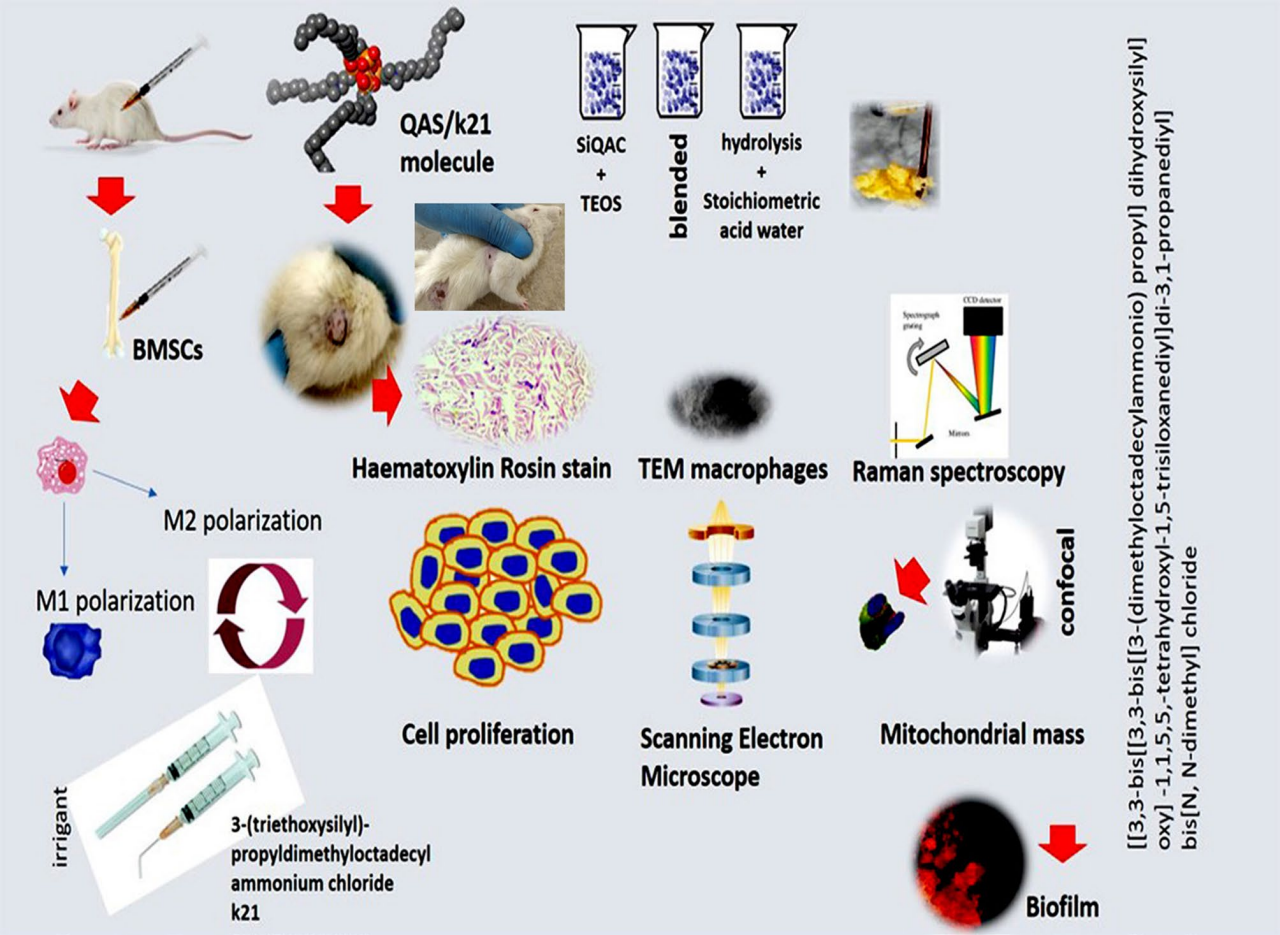
Schematic representation of the animal model utilised in this study with various experiments performed.

Quaternary Ammonium Silane (ethanol-based solution form; k210.5% and 1%) was generously supplied by KHG fiteBac Technology, Marietta, GA, USA. By sol–gel synthesis, the SiQAC molecule was substituted with 3-(triethoxysilyl)-propyldimethyloctadecyl ammonium chloride (i.e., the ethoxy version of SiQAC, abbreviated as Et-SiQAC) and coupled with tetraethoxysilane (Fig. 6). As a result, a fully hydrolyzed, ethanol-soluble QAS molecule that was partially condensed S (1-octadecanaminium, N,N') was formed. -[[3,3-bis[[3,3-bis[[3-(dimethyloctadecylammonio) propyl] dihydroxysilyl] oxy] -1,1,5,5,-tetrahydroxyl-1,5-trisiloxanediyl]di-3,1-propanediyl] bis[N, N-dimethyl] chloride (1:4); CAS no. 1566577–36-3; codenamed k21) (Fig. 7). After complete hydrolysis, infrared spectra (Fig. 6) were obtained, demonstrating the presence of silanol groups and ethanol. The Si–O–Si cyclic open chain species have been well documented. The hydrolysis occurred in more than one alkoxy group, determining the reaction's stability. The OH and O–Si units were attached to a specific (electrophilic) silicon atom, increasing the silanol groups of Si–O–Si bonds and allowing the formation of a three-dimensional network. The use of tetraethoxysilane as anchoring unit for QAS synthesis enables a three-dimensional, organically modified silicate network to be formed, by hydrolysis of remnant silanol groups (Fig. 6) and subsequent condensation of tetra- and triethoxysilane molecules to form additional Si–O–Si linkages. Unlike the condensation reaction of methoxysilanes which produces methanol as a toxic by-product, sol–gel reaction between ethoxysilanes produces ethanol as the condensation product. This enables QAS to be used for intraoral application without purification to removal methanol. The FTIR infrared peaks confirmed incomplete condensation (the peak shape usually gets altered, which was also the case here and the altered peak shifted to higher wavenumbers, since the minimum of the peak is located at higher wavenumbers relative to the peak maximum) which would condense fully on exposure to moisture or dentin.

### Animal models

Ethical approval for this study was obtained from Post Graduate Medical Institute Lahore Institutional Review Board. The present study followed international, national, and/or institutional guidelines for humane animal treatment and complied with relevant legislation from Post Graduate Medical Institute Institutional Review Board. For the animal model evaluation as depicted in Fig. 7, 27 albino wistar rats of either gender weighing between 200 and 250 g were selected with diseased rats excluded from the study. The animals were segregated into 3 groups: control (saline) and two experimental (0.5% and 1%k21) groups (*n* = *3*) for each time interval. Two concentrations were used to compare the efficacy with two different concentrations. The animals were kept safe in pathogen-free iron cages under controlled temperature (25 ± 2 °C) and humidity (55 ± 5%) with a 12-h dark–light cycle*.* The animals were fed standard laboratory chow and water ad libitum. The study was started after one week of acclimatization into the new environment following experimental protocols for the human use of laboratory animals established at Postgraduate Medical Institute (PGMI) Lahore, Pakistan.

### Macrophage derivation

10-week-old male albino wistar rats were euthanized following the same procedure as mentioned. The tibia and femur were collected aseptically and cut in almost half and placed in reaction tube (1.5 ml). Centrifugation was performed (10 min, 5000 × *g*) and cells separated with a cell strainer (40 μm). The bone marrow cells were procured and differentiated using RPMI 1640 culture medium added with 10% heat-inactivated fetal calf serum (FBS, hyclone, SCV30106), 25% L929 fibroblast supernatant and 1.5% penicillin streptomycin. Ten millilitres of the suspension were transferred in 100 × 20 mm bacterial petri dishes (#430,591; Corning, Corning, NY, USA) and cultivated at 37 °C and 5% CO_2_. After 2 days, the plates were washed with phosphate buffered saline solution (PBS) with removal of all non-adherend cells from the supernatant. The attached macrophages were detached using 0.02% EDTA in PBS and incubated for 5 min on ice followed by 1 min at − 20 °C. After resuspension in α-MEM, the cell number was determined with a cell counter (Roche Diagnostics GmbH, Mannheim, Germany) and macrophages seeded and harvested at a density of 1.5 × 10^4^ cells/cm^2^ for further experiments.

### Irrigant exposure

Once the cells had achieved 80% confluency, the cells were trypsinized and seeded at a number of 80, 000 cells per well in a 12-well tissue culture plate (Corning; Corning, NY) using DMEM for irrigant exposure. In preparation for irrigant exposures, 0.1 ml of irrigants were added slowly in the DMEM medium for further experiments.

### Mitochondrial mass detection through mitotracker green

The macrophages of known concentrations were dispensed into FACS tubes at a final number count of 8 × 10^4^–10 × 10^4^ / 200 μL/tube. MitoTracker-Green (MTG, Molecular Probes, M7514) was reconstituted in DMSO at a low concentration introduced after treatment of the cells using different irrigants. The cells were stained with 100 nm MTG, and the mean fluorescence intensity (MFI) on the FL-1 channel was quantified using the FlowJo software (v10.8.1, https://www.flowjo.com/solutions/flowjo^65^, Tree Star, San Jose, CA, USA). The mitochondrial morphology after staining was examined using laser scanning confocal microscopy (LSCM, Olympus, Tokyo, Japan) with mitochondrial mass analyzed using ImageJ software (v 1.8.0, https://imagej.en.softonic.com^66^, NIH, Bethesda, MD) averaging for 300 cells per sample. The magnification changer was set to 1.6 × zoom (pixel size = 0.1667 μm) or 1 × zoom (pixel size = 0.2667 μm).

### Transmission electron microscopy

After treatment using different irrigants, 2 × 10^6^ macrophage cells were fixed in 4% glutaraldehyde (Santa Cruz, CA, USA) at 4 °C for 24 h. The samples were exposed to 1% osmium tetraoxide, and ethanol dried using an ascending series and embedded finally using araldite. Thin sections of 90 nm were stained with lead citrate and uranyl acetate and analysed using a JEM2100 (JEOL, Japan) transmission electron microscope at 200 kV to examine the extracellular vesicle. Nanoparticle tracking analysis was used to characterise the isolated vesicles. The isolated vesicles were imaged using 150 nm spaces with an electron beam passing through them.

### M1/M2 polarization

The bone marrow derived cells were induced differentiation in a mixed medium using L929 cell supernatant, 1-part fetal bovine serum and 6 parts of high glucose DMEM. After one week of growth, the cells were exposed to irrigants for 5 min and eventually polarized to M1 polarization by incubating cells with 5 ng/mL IFN-γ (R&D Systems, USA) with 50 ng/mL LPS (*E. coli* LPS, Sigma Aldrich, MY) for 24 h. For M2 polarization, the cells were incubated with 10 ng/mL IL-4 and 10 ng/mL IL-13 (R&D Systems, USA). The cells were validated by density-gradient centrifugation using Ficoll-Histopaque (Sigma-Aldrich, St Louis, MO), followed by immunomagnetic separation according to manufacturer’s instructions (Dynabeads Untouched Human Monocytes Kit from Invitrogen). The purity of the separation was confirmed measuring CD14 positive cells by flow cytometry (> to 95%) and evaluated using TEM (data not shown). In addition, the IL-6 and IL-1p supernatant were detected from the cell culture by cytometric bead array (CBA) using an LSRFortessa (BD Biosciences, USA). The detection limits were set to 1.6 pg/mL and 0.5 pg/mL for IL-6 and IL-10, respectively.

### Scratch analysis and quantitative polymerase chain reaction (qPCR)

Scratch assay (Romerowicz-Misielak et al., 2021) was used to assess the wound healing effect of 0.5%k21. Murine primary fibroblast (NIH-3T3) cells ((ATCC; VA, USA) were used as cell model. The cells were grown in Dulbecco’s Modified Eagle’s Medium (DMEM) supplemented with 10% v/v fetal bovine serum (FBS;), and 1% v/v Pen-Strep (100 U/mL penicillin and 100 µg/mL streptomycin) in a humidified incubator at 37 °C and 5% CO_2_.

The dose of the test compound, 0.5%k21, for wound healing assay was determined by performing the cytotoxicity studies on 3T3 cells using MTT (3-(4,5-Dimethylthiazol-2-yl)-2,5-diphenyltetrazolium bromide) assay. The cells were treated with k21 for 48 h to determine the cell viability. The cell viability was calculated by measuring the absorbance of the solutions at 550 nm (Reference: 630 nm) using a microplate reader (Tecan Spark; Tecan, Switzerland).

$${\text{Percent cell viability }} = \frac{{A_{{{\text{Treatment}}}} - A_{{{\text{Blank}}}} }}{{A_{{{\text{Vehicle}}}} - A_{{{\text{Blank}}}} }} \times 100$$

The test compound, 0.5%k21, did not show toxic effects at the desired concentration. The same concentration was used to assess its wound healing activity. The cells (2 × 10^6^ cells/well) were seeded in TC-treated 6-well plates and incubated at 37 °C and 5% CO_2_ for 24 h and allow the cells to form a monolayer. A sterile P100 micropipette tip was used to make a linear scratch and the detached cells were removed by washing with phosphate saline buffer (PBS). Then 0.5%k21 test compound was added, and the wound area, and wound closure were observed for 24 h, which are calculated from the photographs of the cells at 24 h and 48 h. The photographs were captured using a Nikon Eclipse Ti-U Inverted microscope (Nikon, Japan). Wound areas were measured using the NIS Elements 3.0 AR software (Nikon, Japan). Wound closure and wound closure were calculated using the formulas adapted from Liang et al. (Liang et al., 2007):

$$Wound \,area \,\left( {mm^{2} } \right) = Width\, \left( {mm} \right) \times Length\, \left( {mm} \right)$$

$$Wound\, closure\, at\, t \,\left( \% \right) = \left[ {\frac{{Migrated\, cell \,surface \,area\, \left( {mm^{2} } \right)}}{{Total \,surface \,area \,\left( {mm^{2} } \right)}}} \right] \times 100\%$$

The mechanisms involved in wound healing activity of 0.5%k21 was determined by measuring the relative expression of the genes in 0.5%k21 treated samples compared to vehicle treated cells. Total ribonucleic acid (RNA) was extracted from cells using the RNeasy Mini Kit (Qiagen, Germany) according to the protocol. The RNA (0.5 µg) was reverse transcribed using the ReverTra ACE qPCR RT Master Mix with gDNA remover (Toyobo, Japan), following the manufacturer’s protocol, with a thermal cycler (Bio-Rad Laboratories, CA, USA). Quantitative polymerase chain reaction (qPCR) was carried out with THUNDERBIRD SYBR qPCR Mix (Toyobo, Japan) using C1000 TOUCH thermal cycler (Bio-Rad Laboratories, CA, USA). The expression of the genes was quantified using CFX96 Real-Time System (Bio-Rad Laboratories, CA, USA). The genes tested in this study were C–C motif chemokine ligand 2 (CCL2), vascular cell adhesion protein 1 (VCAM-1), intercellular adhesion molecule-1 (ICAM-1), C-X-C motif chemokine ligand 10 (CXCL10), C-X-C motif chemokine ligand 11 (CXCL11), C-X-C motif chemokine ligand 8 (CXCL8), collagen I, epidermal growth factor receptor (EGFR), tissue inhibitor of metalloproteinases 2 (TIMP-2), macrophage colony-stimulating factor (M-CSF), and the reference gene, glyceraldehyde 3-phosphate dehydrogenase (GAPDH). The expression of the genes were analyzed using the Bio-Rad CFX Manager software, version 2.1., (https://www.bio-rad.com/en-my/sku/1845000^68^, Bio-Rad Laboratories, CA, USA) and normalized against glyceraldehyde-3-phosphate dehydrogenase (GAPDH) as the reference gene (Sikand et al., 2012). The list of genes and primers used in this study were tabulated in Table 2. The relative gene expression was determined using the 2^-ΔΔCt^ method and expressed in arbitrary units.

### Raman data acquisition

Raman spectroscopy (Horiba Jobin Yvon (Paris, France) of macrophage cells was performed using a 532 nm laser (Sapphire SF-532; Coherent, Santa Clara, CA) excitation and NA/1.35 oil-immersion objective lens focusing the laser beam on the sample. The back scattered Raman signal was passed through the filters and focused on the slit of the spectrograph SP2300; Princeton Instruments, Acton, MA) with 1200 lines/mm grating of the signal light. The sample scaffold was deposited on a glass bottomed dish placed on the Raman x-y-z stage. All Raman spectrums were obtained at room temperature (23 °C) in the same environment with a laser power at 10mW in between 400 and 1800 cm^-1^ in 60 s. All measurements were recorded using WinSpec software and processed using Matlab (The MathWorks, Natick, MA) while preprocessed by the same method. All instrumental response and wavelength positions were calibrated. For Raman spectra, three different locations were selected for each group.

### Biocompatibility analysis

The cell counting kit-8 (CCK8, ApexBio, USA) was used to indirectly measure the cytotoxicity of BMSCs treated with modified medium^31^. BMSCs were seeded at a density of 10^3^ cells per well in a 96-well plate and incubated overnight. After replacing the medium with the prepared modified medium and incubating for 24 h, 10 L CCK-8 assay reagent was added to each well and incubated for 1 h. A Microplate Reader was used to measure the optical density (OD) at 450 nm (Thermo Fisher, America). As a control, cells cultured in complete medium were used. The viability of BMSCs was calculated using the following equation:

1 $${\text{Cell viability }}\left( \% \right) \, = \, \left( {{\text{OD}}_{{{\text{test}}}} - {\text{ OD}}_{{{\text{blank}}}} } \right) \, / \, \left( {{\text{OD}}_{{{\text{control}}}} - {\text{ OD}}_{{{\text{blank}}}} } \right) \, \times { 1}00\%$$

### Immunocytochemistry and fluorescence microscopy

Immunocytochemistry and fluorescence microscopy were used to examine the expression of CD163. In brief, a 12-well plate’s wells were filled with rounded coverslips (12 mm in diameter). Each well received 500 g/l fibronectin (Sigma Aldrich, St Louis, MO, USA) and was incubated for 12 h at 4 °C.

### Scanning electron microscopy of bacterial cells

Control and experimental teeth were extracted from rats and stored in 10% formalin for 72 h. Three rats of each group were selected and anesthetized with Ketamine and Xylazine with respect to their weights. Three dentin slabs were obtained from the mid coronal part of each tooth. The slabs were wet polished with increasing grit sizes of SiC papers (Carbimet, Buehler, Lake Bluff, USA) and thoroughly rinsed with deionized water for 10 min with continuous ultrasonication.

SEM was used to investigate the interaction of disinfectants with *E. faecalis*. *Enterococcus faecalis* (ATCC 29,212) was grown in Brain Heart Infusion broth (BHI; Difco Laboratories, Detroit, MI, USA) and adjusted to 1.5 McFarland turbidity. Bacteria were cultured anaerobically on blood agar plates at 37 °C for 20 h. The bacterium was then transferred to brain heart infusion (BHI) broth supplemented with 8% sucrose (pH 7.4) and a trace of xylitol (0–2%) for 48 h at 37 °C. The cells were then centrifuged for 15 min at 4000 rpm, and the cell pellets were washed three times with phosphate buffered solution (PBS, 0.01 M, pH 7.2). The cells were suspended in 100 mL of growth medium and adjusted to McFarland standard no. 3 concentration (10^9^ cells/mL). 5 ml of BHI broth and bacterial inoculum were used to fill the canals with sterilising syringes and allowed to react for 3 days. After 3 days, the irrigation protocol was followed. Each tooth was dried under aseptic conditions with sterile paper points. Parallel grooves (2) were made on external surfaces across the mesio-distal direction, allowing a split fracture to be performed by a single operator. The roots were then separated with a chisel and a hammer, and the teeth were collected for scanning electron microscopy.

The dentin specimens were pre-fixed for 30 min in 2.5% glutaraldehyde. The specimens were washed in PBS and post-fixed for 2 h in 1% osmium tetraoxide. The post-fixed cells were dehydrated using ascending grades of ethanol solutions after being washed with PBS (50, 70, and 90 ethanol). The specimens were finally dried at 37 °C before being mounted on stubs for gold sputtering for 120 secs under vacuum in a Philips/FEI XL30 FEG (Hisboro, OR USA) at a 10 kV accelerating voltage.

### Confocal analysis

*Enterococcus faecalis* (ATCC 29,212) was grown in Brain Heart Infusion broth (BHI; Difco Laboratories, Detroit, MI, USA) to 1.5 McFarland standard turbidity. After growth on blood agar plates, the bacteria were transferred in BHI with 8% supplemental sucrose and 2% xylitol (pH 7.4) at 37 °C for 48 h. The cells were washed with PBS and cells suspended in 100 ml of growth medium and adjusted to a McFarland concentration standard no. 3 (110 cells/ml). In preparation for irrigant exposures, 0.1 ml of irrigants were added slowly in the DMEM medium for further experiments.

To distinguish viable and non-viable bacteria, confocal microscopy was performed. All confocal images were collected using Leica TCS SP5 hardware with DM600 fixed stage with emission profiles for SYTO^R^ 9 stains measured at 500 to 550 nm. Excitation was performed argon 488 nm laser with the power set to 20% and maximum intensity of 50%. The scanning speed was set at 800 Hz and 512 × 512-pixel resolution. The bacteria were examined on 42 × 0.17 mm slides covered with cover slips. All z-stacks were performed with a z-step size of 5.98 μm with line average set to 1 and stack images performed in triplicates. The captured images were analysed using Bitplane Imaris software version 6.3.1 (Bitplane A.G. https://www.swissbiotech.org/listing/bitplane-ag/^67^) by converting the images into Bitplan Imaris. The spot detection was measured at 5 μm as the clipping plane was used to obtain a cross section view in a 3D image.

### Statistical analysis

The analyses were done using SPSS software version 20.0 (IBM, Chicago-USA). For comparison of ordinal data among 3 groups, Kruskal Wallis test of k-independent variables. Wilcoxon Mann–Whitney U test was used for pairwise comparison between control and experimental groups. Wilcoxon Sign-rank test was used for comparison of within group effect at 2 h vs. 2 days vs. 2 weeks for histological analysis (data not shown). Unless stated otherwise, the *p* value for all statistical tests were stated as *p* < *0.05* as all datasets were subjected for homoscedasticity and normality. Where datasets were normally distributed, a one-way ANOVA was performed followed by Tukey post-hoc comparison.

### Ethical approval

The ethical committee at International Medical University approved the study, protocol number IMU 259/2020. All authors signed the consent forms. The reporting in the manuscript follows the recommendations in the ARRIVE guidelines.

## Supplementary Information

Supplementary Information.

## References

[1] Dunavant TR, Regan JD, Glickman GN, Solomon ES, Honeyman AL (2006). Comparative evaluation of endodontic irrigants against Enterococcus faecalis biofilms. J. Endod..

[2] Shen Y, Stojicic S, Haapasalo M (2011). Antimicrobial efficacy of chlorhexidine against bacteria in biofilms at different stages of development. J. Endod..

[3] Torabinejad M, Handysides R, Khademi AA, Bakland LK (2002). Clinical implications of the smear layer in endodontics: a review. Oral Surg. Oral Med. Oral Pathol. Oral Radiol. Endod..

[4] Haapasalo M, Endal U, Zandi H, Coil JM (2005). Eradication of endodontic infection by instrumentation and irrigation solutions. Endod. Top..

[5] Umer D, Abhishek P, Jukka M, Cynthia Y, Hany Mohamed AA, Amr F (2020). Properties of a modified quaternary ammonium silane formulation as a potential root canal irrigant in endodontics. Dent. Mater..

[6] Gutarts R, Nusstein J, Reader A, Beck M (2005). In vivo debridement efficacy of ultrasonic irrigation following hand-rotary instrumentation in human mandibular molars. J. Endod..

[7] Zhang C, Du J, Peng Z (2005). Correlation between Enterococcus faecalis and persistent intraradicular infection compared with primary intraradicular infection: a systematic review. J. Endo..

[8] Saleh IM, Ruyter IE, Haapasalo M, Orstavik D (2010). Survival of Enterococcus faecalis in infected dentinal tubules after root canal filling with different root canal sealers in vitro. Inter. Endo. J..

[9] Parisi L, Gini E, Baci D, Tremolati M, Fanuli M, Bassani B (2018). Macrophage polarization in chronic inflammatory diseases: killers or builders?. J. Immunol. Res..

[10] Shapouri-Moughadam A, Mohammadian S, Vazini H, Taghadosi M, Esmaeili SA, Mardani F (2018). Macrophage plasticity, polarization, and function in health and disease. J. Cell. Physiol..

[11] Liu YC, Zou XB, Chai YF, Yao YM (2014). Macrophage polarization in inflammatory diseases. Int. J. Biol. Sci..

[12] Zhou D, Huang C, Lin Z, Zhan S, Kong L, Fang C (2014). Macrophage polarization and function with emphasis on the evolving roles of coordinated regulation of cellular signaling pathways. Cell. Signal..

[13] Krzyszczyk P, Schloss R, Palmer A, Berthiaume F (2018). The role of macrophages in acute and chronic wound healing and interventions to promote pro-wound healing phenotypes. Front. Physiol..

[14] Bashir S, Sharma Y, Elahi A (2016). Macrophage polarization: the link between inflammation and related diseases. Inflamm. Res..

[15] Folmes CDL, Dzeja PP, Nelson TJ (2012). Mitochondria in control of cell fate. Circ. Res..

[16] Jin HS, Suh HW, Kim SJ (2017). Mitochondrial control of innate immunity and inflammation. Immune. Netw..

[17] Simons K, Ehehalt R (2002). Cholesterol, lipid rafts, and disease. J. Clin. Invest..

[18] Liu J, Huan C, Chakraborty M (2009). Macrophage sphingomyelin synthase 2 deficiency decreases atherosclerosis in mice. Circ. Res..

[19] Daood U, Yiu CKY, Burrow MF, Niu LN, Tay FR (2017). Effect of a novel quaternary ammonium silane cavity disinfectant on durability of resin-dentine bond. J. Dent..

[20] Umer D, Yiu CK, Burrow MF, Niu LN, Tay FR (2017). Effect of a novel quaternary ammonium silane on dentin protease activities. J. Dent..

[21] Umer D, Matinlinna JP, Pichika MR, Mak KK, Nagendrababu V, Fawzy AS (2020). A quaternary ammonium silane antimicrobial triggers bacterial membrane and biofilm destruction. Sci. Rep..

[22] Turova NY, Turevskaya EP, Kessler VG, Yanovskaya MI (2002). The Chemistry of Metal alkoxides.

[23] Danks AE, Hall SR, Schnepp Z (2016). The evolution of ‘sol–gel’ chemistry as a technique for materials synthesis. Mater. Horiz..

[24] Daood U, Burrow MF, Yiu CKY (2020). Effect of a novel quaternary ammonium silane cavity disinfectant on cariogenic biofilm formation. Clin. Oral Investig..

[25] Tischer M, Pradel G, Ohlsen K, Holzgrabe U (2012). Quaternary ammonium salts and their antimicrobial potential: targets or nonspecific interactions?. Chem. Med. Chem..

[26] Daood U, Balasankar MP, Ibrahim MA, Pichika MR, Mak K-K, Fawzy AS (2021). PLGA nanoparticles loaded with quaternary ammonium silane and riboflavin for potential applications in adhesive dentistry. Int. J. Ad. Adhesv..

[27] Daood U, Parolia A, Elkezza A, Yiu CK, Abbot P, Matinlinna JP, Fawzy AS (2019). An in vitro study of a novel quaternary ammonium silane endodontic irrigant. Dent. Mater..

[28] Daood U, Yiu CKY (2019). Transdentinal cytotoxicity and macrophage phenotype of a novel quaternary ammonium silane cavity disinfectant. Dent. Mater..

[29] Oishi Y, Manabe I (2018). Macrophages in inflammation, repair, and regeneration. Int. Immunol..

[30] Orihuela R, McPherson CA, Harry GJ (2016). Microglial M1/M2 polarization and metabolic states. Br. J. Pharmacol..

[31] Zhu Y, Herndon JM, Sojka DK, Kim KW, Knolhoff BL, Zuo C, Cullinan DR, Luo J, Bearden AR, Lavine KJ, Yokoyama WM, Hawkins WG, Fields RC, Randolph GJ, DeNardo DG (2017). Tissue-resident macrophages in pancreatic ductal adenocarcinoma originate from embryonic hematopoiesis and promote tumor progression. Immunity.

[32] Meyer A, Laverny G, Bernardi L (2018). Mitochondria: an organelle of bacterial origin controlling inflammation. Front. Immunol..

[33] Jain N, Vogel V (2018). Spatial confinement downsizes the inflammatory response of macrophages. Nat. Mater..

[34] Fonseca TB, Sanchez-Guerrero A, Milosevic I (2019). Mitochondrial fission requires DRP1 but not dynamins. Nature.

[35] Piantadosi CA, Suliman HB (2012). Transcriptional control of mitochondrial biogenesis and its interface with inflammatory processes. Biochim. Biophys. Acta..

[36] Murphy MP, Smith RA (2007). Targeting antioxidants to mitochondria by conjugation to lipophilic cations. Annu. Rev. Pharmacol. Toxicol..

[37] Datta S, Baudouin C, Brignole-Baudouin F, Denoyer A, Cortopassi GA (2017). The eye drop preservative benzalkonium chloride potently induces mitochondrial dysfunction and preferentially affects LHON mutant cells. Invest. Ophthalmol. Vis. Sci..

[38] Visers MC, Thomas C (1997). Hypochlorous acid disrupts the adhesive properties of subendothelial matrix. Free Radical Biol. Med..

[39] Jiménez-Rubio A, Segura JJ (1998). The effect of the bleaching agent sodium perborate on macrophage adhesion in vitro: implication in external cervical root resorption. J. Endodontics..

[40] Vi L (2015). Macrophages promote osteoblastic differentiation in-vivo: implications in fracture repair and bone homeostasis. J. Bone Miner. Res..

[41] Cappariello A, Loftus A, Muraca M, Maurizi A, Rucci N, Teti A (2018). Osteoblast derived extracellular vesicles are biological tools for the delivery of active molecules to bone. J. Bone Miner. Res..

[42] Curtale G, Rubino M, Locati M (2019). MicroRNAs as molecular switches in macrophage activation. Front. Immunol..

[43] Roszer T (2015). Understanding the mysterious M2 macrophage through activation markers and effector mechanisms. Mediat. Inflamm..

[44] Zhu Z, Ding J, Ma Z, Iwashina T, Tredget EE (2017). Alternatively activated macrophages derived from THP-1 cells promote the fibrogenic activities of human dermal fibroblasts. Wound Repair Regen Off Publ. Wound Heal Soc. Eur. Tissue Repair Soc..

[45] Wang YZ, Chen ZQ, Luo GX, He WF, Xu KG, Xu R (2016). In-situ-generated vasoactive intestinal peptide loaded microspheres in mussel-inspired polycaprolactone nanosheets creating spatiotemporal releasing microenvironment to promote wound healing and angiogenesis. ACS Appl. Mater. Interfaces..

[46] Abraham G, Colonno RJ (1984). Many rhinovirus serotypes share the same cellular receptor. J. Virol..

[47] Levin IW, Thompson TE, Huang C (1985). Two types of hydrocarbon chain interdigitation in sphingomyelin bilayers. Biochemistry.

[48] Lamba OP, Borchman D, Lou MF (1991). Structure, and molecular conformation of anhydrous and of aqueous sphingomyelin bilayers determined by infrared and Raman spectroscopy. J. Mol. Struct..

[49] Lallemand T, Rouahi M, Swiader A, Grazide MH, Geoffre N, Alayrac P, Recazens E, Coste A, Salvayre R, Nègre-Salvayre A, Augé N (2018). NSMase2 (type 2-neutral sphingomyelinase) deficiency or inhibition by GW4869 reduces inflammation and atherosclerosis in Apoe −/− mice. Arterioscler. Thromb. Vasc. Biol..

[50] Zhou K, Blom T (2015). Trafficking and functions of bioactive sphingolipids: lessons from cells and model membranes. Lipid Insights..

[51] Kunz TC, Kozjak-Pavlovic V (2019). Diverse facets of sphingolipid involvement in bacterial infections. Front. Cell Dev. Biol..

[52] VanMeer G, Lisman Q (2002). Sphingolipid transport: rafts and translocators. J. Biol. Chem..

[53] Tafesse FG, Rashid Farrokhi A, Schmidt FI, Freinkman E, Dougan S, Dougan M, Esteban A, Maruyama T, Strijbis K, Ploegh HL (2015). Disruption of sphingolipid biosynthesis blocks phagocytosis of Candida albicans. PLoS Pathog..

[54] Umer D, Ranjeet AB, Preena S, Muhammed SI, Abdul KS, Kit-Kay M, Malikarjuna RP, Venkateshbabu N, Ove P (2021). Antibacterial and antibiofilm efficacy of k21-E in root canal disinfection. Dent. Mater. In Press Corrected Proof.

[55] Wynn TA, Chawla A, Pollard JW (2013). Macrophage biology in development, homeostasis, and disease. Nature.

[56] Webb JS, Thompson LS, James S, Charlton T, Tolker-Nielsen T, Koch B (2003). Cell death in Pseudomonas aeruginosa biofilm development. J. Bacteriol.

[57] Ye WH, Fan B, Purcell W, Meghil MM, Cutler CW, Bergeron BE (2018). Anti-biofilm efficacy of root canal irrigants against in-situ Enterococcus faecalis biofilms in root canals, isthmuses, and dentinal tubules. J. Dent..

[58] Foulkes DM (1973). Some toxological observations in chlorhexidine. J. Periodontal. Res. Suppl..

[59] Liu R, Xu Y, Wu D, Sun Y, Gao H, Yuan H (2004). Comparative study on the hydrolysis kinetics of substituted ethoxysilanes by liquid-state 29Si NMR. J. Non Cryst. Solids.

[60] Diogo P, Fernandes C, Caramelo F, Mota M, Miranda IM, Faustino MA, Uliana MP, Santos JM, Gonçalves T (2017). Antimicrobial photodynamic therapy against endodontic *Enterococcus faecalis* and *Candida albicans* mono and mixed biofilms in the presence of photosensitizers: A comparative study with classical endodontic irrigants. Front. Micro..

[61] Gomes BP, Pinheiro ET, Jacinto RC (2008). Microbial analysis of canals of root-filled teeth with periapical lesions using polymerase chain reaction. J. Endod..

[62] Haapasalo M, Orstavik D (1987). In vitro infection and disinfection of dentinal tubules. J. Dent. Res..

[63] Chavez de Paz LE, Bergenholtz G, Dahlen G, Svensater G (2007). Response to alkaline stress by root canal bacteria in biofilms. Int. Endod. J..

[64] Shin SJ, Jee SW, Song JS (2008). Comparison of regrowth of Enterococcus faecalis in dentinal tubules after sealing with gutta-percha or Resilon. J. Endod..

[65] https://www.flowjo.com/solutions/flowjo.

[66] https://imagej.en.softonic.com.

[67] https://www.swissbiotech.org/listing/bitplane-ag//

[68] https://www.bio-rad.com/en-my/sku/1845000

