# Supplementary material for: Biochemical changes and macrophage polarization of a silane-based endodontic irrigant in an animal model
Source: Sci Rep. 2022 Apr 15;12:6354. doi: 10.1038/s41598-022-10290-0 (PMC9012771; doi:10.1038/s41598-022-10290-0)
Supplement: Supplementary file 1 — Supplementary Information. [file 41598_2022_10290_MOESM1_ESM.pdf]

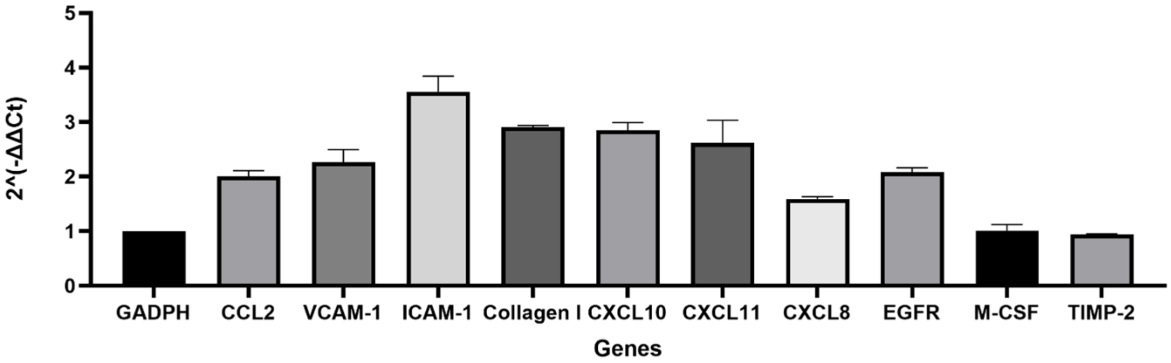

Supplementary Figure 1: Quantitative polymerase chain reaction (qPCR) for gene expression of wound healing. The relative mRNA expression of CCL2, VCAM-1, ICAM-1, Collagen I, CXCL10, CXCL11, CXCL8, EGFR, M-CSF and TIMP-2 in NIH-3T3 cells after 48-hours treatment with Rutin, with respect to negative control. Note: CCL2, C-C motif chemokine ligand 2; VCAM-1, vascular cell adhesion protein 1; ICAM-1, intercellular adhesion molecule-1; CXCL10, C-X-C motif chemokine ligand 10; CXCL11, C-X-C motif chemokine ligand 11 and CXCL8, C-X-C motif chemokine ligand 8; EFGR, epidermal growth factor receptor; M-CSF, macrophage colony-stimulating factor; TIMP-2, tissue inhibitor of metalloproteinases 2
